# OATrack: A Quality-Gated Progressive Association Framework with a YOLO Detection Cache for UAV Small-Object Multi-Object Tracking

**DOI:** 10.3390/s26165222

**Published:** 2026-08-18

**Authors:** Lei Qi, Liejun Wang, Shaochen Jiang

**Affiliations:** School of Computer Science and Technology, Xinjiang University, Urumqi 830046, China; qilei@xju.edu.cn (L.Q.); scj@xju.edu.cn (S.J.)

**Keywords:** UAV vision, small-object tracking, multi-object tracking, tracking-by-detection, quality-gated association, identity preservation, TrackEval

## Abstract

Unmanned aerial vehicle (UAV) multi-object tracking (MOT) is challenging because small targets produce unstable confidence and localization, while viewpoint changes and short occlusions make association ambiguous. We present OATrack, an integrated association framework tailored to UAV small-object tracking. OATrack combines detection–track pair quality based on detection reliability and current-state geometric consistency, quality-gated track-state protection with a track-specific scale prototype, and three-stage progressive assignment for active-track matching and recently lost-track recovery. A standardized YOLO11m-smallobj detection cache supplies identical detections to all association methods and enables reproducible fixed-input evaluation. On VisDrone-MOT val, OATrack reaches HOTA 38.5847 versus 38.4797 for official Ultralytics ByteTrack while reducing identity switches (IDSW) by 69.2%, track fragmentation by 29.7%, and false positives by 68.8%. On the complete 20-sequence UAVDT test split, OATrack improves HOTA, MOTA, and IDF1 from 41.4345, 7.5162, and 51.4916 to 42.7743, 24.8760, and 53.2829, respectively, and reduces IDSW from 639 to 295. HOTA-component and size/occlusion analyses further demonstrate stronger association consistency and false-positive control across the two UAV benchmarks.

## 1. Introduction

Unmanned aerial vehicle (UAV) vision sensors provide flexible deployment, wide-area coverage, and variable observation viewpoints. They are therefore becoming a practical sensing component in low-altitude intelligent perception systems. In traffic monitoring, UAVs can observe intersections, arterial roads, overpasses, parking areas, and congested road segments from elevated viewpoints. In public-safety and emergency-response tasks, they can also acquire large-scale dynamic imagery for object localization, behavior analysis, and resource dispatching. For such continuous videos, multi-object tracking (MOT) aims to localize multiple objects in each frame while preserving their identities over time. It is a key basis for trajectory analysis, speed estimation, behavior understanding, and spatio-temporal event reasoning. UAV tracking benchmarks such as VisDrone and UAVDT have further highlighted the importance and difficulty of this setting [1,2].

**Figure 1:**
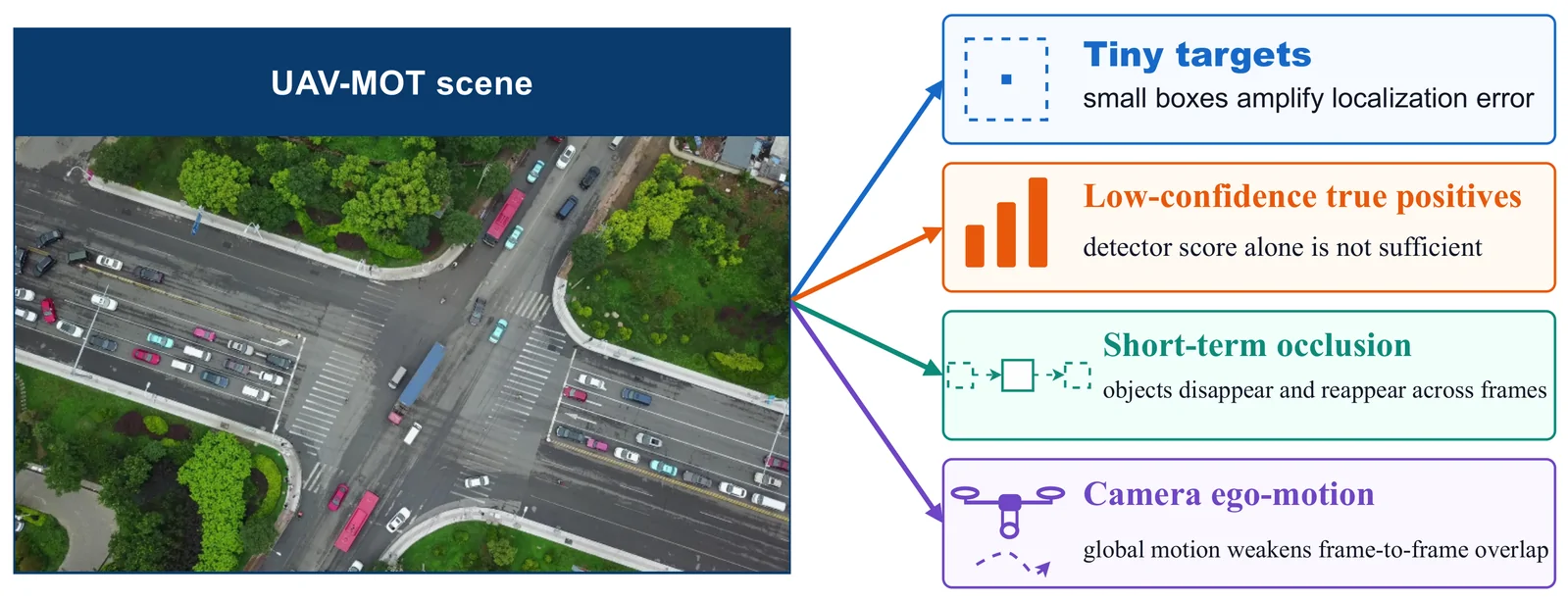
Typical challenges in UAV small-object multi-object tracking. Aerial viewpoints produce small-object scales and unstable detection confidence, while short-term occlusion and camera motion weaken frame-to-frame association reliability and may cause missed associations, track interruptions, and identity switches.

Compared with videos from fixed ground cameras, UAV MOT concentrates several difficulties in the same sequence. Pedestrians, vehicles, and cyclists often occupy only a small number of pixels in aerial views, leaving weak local texture and unstable appearance cues. UAV ego-motion introduces global viewpoint changes, so the same object may undergo large position and scale shifts between adjacent frames, weakening the reliability of local intersection-over-union (IoU) matching. In dense traffic or crowded scenes, short-term occlusion, crossing motion, and background interference further increase track fragmentation and identity switches. Figure 1 summarizes typical challenges in UAV small-object MOT, including small-object scale, confidence fluctuation, short-term occlusion, and camera motion.

Existing MOT methods can be broadly grouped into joint detection-tracking methods and tracking-by-detection methods. End-to-end Transformer or query-based trackers model detection and tracking within a unified framework, and representative designs include TransTrack, TrackFormer, and MOTR  [3,4,5]. However, such models often require higher training cost, more complex engineering, and sufficient task-specific data. Tracking-by-detection decouples detection and association, which makes the detector and tracker easier to optimize separately and supports reproducible engineering pipelines. SORT established a compact online tracking framework based on Kalman filtering and Hungarian assignment [6]. DeepSORT added appearance re-identification to reduce identity switches after occlusion [7]. ByteTrack improved recall by associating low-score detections [8]. BoT-SORT incorporated camera-motion compensation and ReID into a robust association framework [9], while OC-SORT revisited observation-centered motion consistency for robust online tracking [10]. These methods provide important references for designing association strategies in UAV small-object MOT.

However, directly applying general MOT methods to UAV small-object videos remains challenging. Low-confidence detections may be either true distant objects or background noise. If detections are filtered only by score, true small targets can be discarded; if the threshold is reduced without constraints, false positives and erroneous associations may propagate. ReID-based methods are also affected by small crop size and weak texture. These observations motivate an association design that evaluates the reliability of a detection relative to a track, protects auxiliary track state from unreliable evidence, and handles active and recently lost tracks progressively without modifying the detector backbone.

Building on confidence-aware association, observation-centered motion modeling, and quality-aware track management [8,9,10], OATrack focuses on the interaction between unreliable UAV detections and track-specific association evidence. It evaluates each detection relative to a candidate track, protects the track’s scale state from low-quality observations, and resolves strict matching, relaxed matching, and lost-track recovery in a progressive sequence. This integrated design targets the unstable scores, weak appearance cues, and rapid geometric changes that characterize UAV small-object videos. A standardized YOLO detection interface further decouples detector output from association, enabling deterministic replay and fixed-input comparison across tracking methods.

The main contributions of this paper are as follows:

1. We formulate detection–track pair quality from detection reliability and geometric consistency with the current track state. Unlike confidence-only partitioning, the gate asks whether a particular detection is plausible for a particular track.
2. We combine pair-quality gating with a protected track-specific scale prototype whose updates are controlled by match quality, reducing state contamination from unstable small-object detections.
3. We organize assignment into strict active-track matching, relaxed matching of the remaining active tracks and detections, and recently lost-track recovery. The stages use the same pair-quality matrix with progressively lower acceptance thresholds.
4. We establish a reproducible fixed-input evaluation protocol through a standardized detection cache and provide comprehensive comparisons with official Ultralytics ByteTrack, including HOTA components, object-size/occlusion analyses, per-sequence results, and runtime decomposition.

## 2. Related Work

### 2.1. UAV Vision Datasets and Small-Object MOT

VisDrone and UAVDT are widely used benchmarks for UAV object detection and tracking [1,2]. VisDrone contains multiple object categories, including pedestrians, vehicles, and bicycles, and is characterized by small objects, dense scenes, and diverse aerial viewpoints. UAVDT focuses on vehicle detection and tracking from UAV videos and includes different flying altitudes, camera angles, occlusion states, and weather conditions. Both datasets reflect the coexistence of small-object scale, camera motion, and scale variation in UAV vision.

The central difficulty of UAV MOT is that detection errors are amplified in the association stage. Pixel-level localization noise in a small bounding box can sharply change IoU, so a detection and a track that should match may appear geometrically inconsistent. At the same time, a low detection score does not necessarily imply background noise, especially for distant vehicles, pedestrians, and partially occluded targets. Raising the detection threshold may reduce false positives but increase false negatives and track breaks; lowering it may improve recall but introduce more false detections and ambiguous associations. UAV MOT therefore depends not only on detector quality but also on association mechanisms that distinguish useful low-score candidates from noise.

### 2.2. Tracking-by-Detection Multi-Object Tracking

SORT models object states with a linear motion process, predicts the next-frame location with a Kalman filter, and builds the assignment cost from IoU [6]. It is simple and fast, but identity switches are likely under occlusion or long-term disappearance. DeepSORT extends SORT with appearance embeddings and combines geometry with ReID distance [7]. In UAV small-object scenes, however, object crops are often too small for stable appearance discrimination.

Other influential online trackers couple detection and association more tightly. Tracktor reuses detector regression for frame-to-frame localization, CenterTrack predicts object centers and inter-frame offsets, FairMOT jointly learns detection and ReID features, and QDTrack learns quasi-dense similarity for association [11,12,13,14]. These designs show that detection quality, object localization, and association evidence are strongly coupled in practical MOT systems.

ByteTrack introduced a practical two-pass strategy that first matches high-score detections and then uses lower-score detections to recover tracks [8]. Its official Ultralytics implementation combines confidence thresholds, Kalman-state prediction, IoU-based costs, and score fusion, and serves as the principal controlled baseline in this study. OC-SORT reconstructs observation-centered trajectories and uses observation-centric momentum to reduce error accumulation after occlusion [10], while BoT-SORT combines Kalman motion, camera-motion compensation, and optional ReID [9]. OATrack complements these approaches with detection–track pair-quality gating, three progressively relaxed acceptance stages, and a quality-protected track-specific scale state designed for small UAV targets.

### 2.3. Quality-Aware Association and Track Memory

Recent MOT research increasingly considers detection quality, trajectory quality, and historical states during association. Detection confidence is frequently used as a candidate filter, but the score is not equivalent to association reliability. OATrack therefore separates detection-only eligibility from pair-level reliability: confidence determines whether a box may enter association, while center-distance and IoU consistency determine whether it is plausible for a particular active or lost track.

Track memory is also common in MOT. Trackers may maintain historical positions, velocities, appearance prototypes, or tracklet information to recover identities after occlusion. OATrack maintains the latest observed box, an exponentially updated per-track scale prototype, and a bounded short-term state history. The current assignment cost uses the observed box and protected scale prototype, while the bounded history records motion and quality statistics for analysis and extensibility. This lightweight state design avoids dependence on appearance embeddings extracted from extremely small-object crops.

### 2.4. Recent Multi-Object Tracking Models

Recent MOT studies have explored faster Transformer tracking, state-space models, diffusion-based motion prediction, anchor-based queries, and tracklet-confidence enhancement. Query-based trackers such as TransTrack, TrackFormer, and MOTR established representative joint detection-tracking formulations with Transformer architectures [3,4,5]. FastTrackTr improves the efficiency of Transformer-based MOT in real-time settings [15]. MambaTrack and SportMamba investigate state-space models for nonlinear motion patterns [16,17]. BoostTrack++ uses tracklet information to improve the use of detections [18]. DiffMOT applies a diffusion-based predictor to nonlinear MOT motion modeling [19]. ABQ-Track introduces anchor-based queries and template matching for Transformer-based joint detection and tracking [20]. SocialTrack further explores social-behavior-inspired mechanisms for complex UAV traffic scenes [21].

These methods have advanced MOT from traditional motion and association models toward richer temporal modeling, detection-quality use, and scene-aware reasoning. Because detector choice, data split, evaluation code, and postprocessing all influence MOT performance, this study uses a unified data-conversion pipeline, a fixed detector-output interface, and TrackEval. The resulting protocol isolates association behavior, supports deterministic replay, and enables a controlled comparison between OATrack and official Ultralytics ByteTrack. Results reported by other published methods provide broader context for UAV and traffic-scene tracking.

### 2.5. Evaluation Metrics

Common MOT metrics include Multi-Object Tracking Accuracy (MOTA), identification F1 score (IDF1), Higher Order Tracking Accuracy (HOTA), identity switches (IDSW), fragments (Frag), false positives (FP), false negatives (FN), and frames per second (FPS). MOTA aggregates FP, FN, and IDSW and is sensitive to detection errors [22]. IDF1 measures identity consistency between predicted and ground-truth trajectories [23]. HOTA jointly considers detection and association accuracy and offers a balanced view of tracking quality [24]. IDSW and Frag indicate identity switches and track fragmentation, respectively, and FPS measures runtime speed. We use TrackEval as the unified evaluation toolkit [25].

## 3. Method

### 3.1. Overall Framework

**Figure 2:**
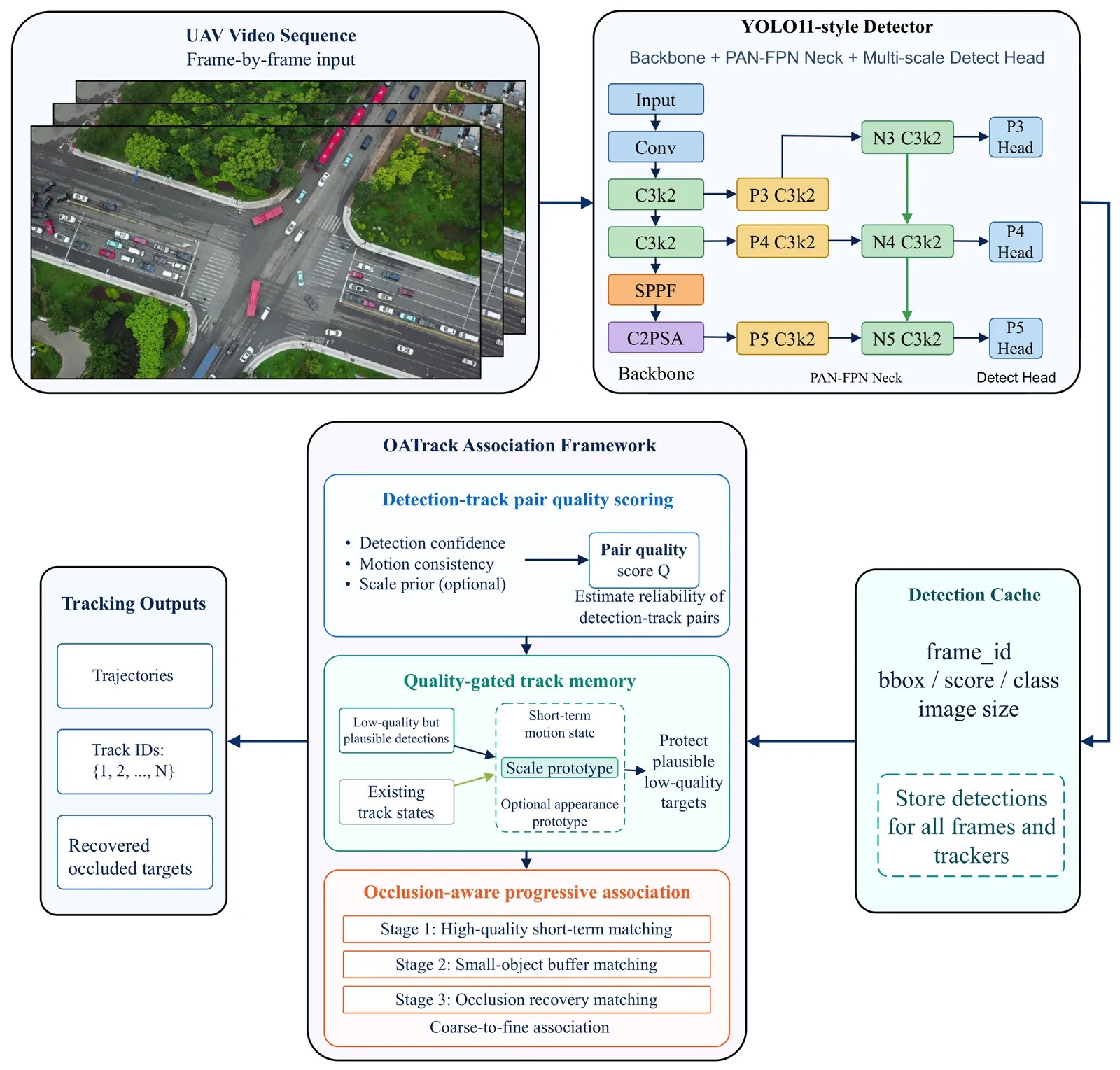
Overall detector–cache–association workflow. The standardized YOLO detection interface supplies fixed detector outputs to the association stage. OATrack combines confidence–motion pair quality, the latest observed state, a protected scale prototype, and three-stage progressive assignment. The global scale-quality prior and appearance interface are optional extensions; the main configuration follows the solid association path shown in the diagram.

OATrack follows the tracking-by-detection paradigm. Figure 2 presents the complete detector–cache–association workflow. A YOLO11m-smallobj model produces bounding boxes, class labels, confidence scores, and image dimensions, which are serialized once and supplied unchanged to every tracker. Starting from this standardized interface, OATrack filters eligible detections, computes detection–track pair quality, applies a track-specific scale-consistency factor, and solves three constrained assignment stages.

During tracking, OATrack maintains active- and recently lost-track sets. Pair quality is computed between current detections and the latest observed boxes and is then adjusted by a track-specific scale prototype. Stage 1 accepts strict active-track assignments; Stage 2 retries unmatched active tracks and detections with a lower threshold; and Stage 3 reconnects remaining detections to recently lost tracks. New tracks require a separate detection-quality threshold, and unmatched tracks are retained for a bounded lost duration. Results are written in the MOTChallenge format and evaluated with TrackEval.

### 3.2. Standardized Detection Interface

The detector is a YOLO11m model trained with the small-object-oriented data configuration, 1536-pixel input size, and the fixed weight file used throughout this study [26]. For each frame, it predicts boxes, class IDs, confidence scores, and image dimensions. The tracker-agnostic serialized record is a standardized interface between detection and association.

The standardized cache decouples detection from association and guarantees that official ByteTrack, the local score–IoU baseline, and OATrack receive byte-identical detector outputs. It also avoids repeated inference and removes detector and non-maximum-suppression variation from the association comparison. During online operation, the same standardized record can be streamed directly in memory.

Each detection-cache record corresponds to one detected object in a frame and stores the standardized fields listed in Table 1:

In the tracking stage, OATrack reads only these records and combines them with existing track states. The cache also supports deterministic replay, per-sequence diagnosis, and controlled parameter analysis. No ground-truth information is stored in or exposed through the cache.

### 3.3. Small-Object Quality-Gated Scoring

Detection confidence is a direct indicator of candidate reliability, but in UAV small-object scenes it cannot fully represent association quality. A low-confidence detection may correspond to a distant small object, a partially occluded object, or a motion-blurred object. Conversely, a high-confidence detection that is spatially inconsistent with the track state may still cause an erroneous match. OATrack therefore builds quality-gated scores at two levels: candidate detection quality and detection–track pair quality.

For a detection *d*, let its width and height be *w* and *h*, the image size be *W* and *H*, and the detection confidence be *s*. The basic confidence quality is

(1) $$q_{\mathrm{conf}}\left(d\right)=s$$

Because small-object scale variation can affect detection reliability, we also retain an optional global scale prior for controlled analysis. The normalized object area is

(2) $$a=\frac{w\cdot h}{W\cdot H}$$

Let $\mu_{s}$ and $\sigma_{s}$ denote the center and spread of a reference log-area distribution. The scale-consistency term is

(3) $$q_{\mathrm{scale}}\left(d\right)=\exp\left(-\frac{{\left(\log\left(a+\epsilon \right)-\mu_{s}\right)}^{2}}{2\sigma_{s}^{2}+\epsilon }\right)$$

This optional term measures consistency with a global small-object reference. In the controlled component experiment, the fixed engineering values are $\mu_{s}=\log\left(0.001\right)$ and $\sigma_{s}=1.0$; they are neither learned by the detector nor optimized by gradient descent. Because one global distribution is not reliable across classes, altitudes, and viewpoints, the final OATrack configuration disables this term.

When the scale prior is enabled, detection quality is defined as

(4) $$q_{\det}\left(d\right)=\sigma \left(\alpha \;\mathrm{logit}\left(q_{\mathrm{conf}}\left(d\right)\right)+\beta \;\mathrm{logit}\left(q_{\mathrm{scale}}\left(d\right)\right)+b\right)$$

where σ(·) is the sigmoid function. The coefficients are fixed configuration values. The global-scale component analysis uses α=1, β=1, and b=0. The main OATrack configuration uses α=1, β=0, and b=0; therefore, the identity σ(logit(s))=s reduces the detection-quality term to $q_{\det}=s$.

For a detection–track pair (d,t), OATrack evaluates spatial consistency between the detection box and the latest observed track box. Let $b_{d}$ be the detection box, $b_{t}$ be the current state box, and $\Delta_{c}$ be their center distance normalized by the square root of the track-box area. The geometric consistency term is defined as

(5) $$q_{\mathrm{motion}}\left(d,t\right)=\lambda \exp\left(-0.5\Delta_{c}^{2}\right)+\left(1-\lambda \right)\mathrm{IoU}\left(b_{d},b_{t}\right)$$

The final detection–track pair quality is

(6) $$Q\left(d,t\right)=q_{\det}{\left(d\right)}^{\gamma }\cdot q_{\mathrm{motion}}{\left(d,t\right)}^{1-\gamma }$$

where γ balances detection reliability and geometric consistency. Before the pair matrix is built, detections must satisfy s≥min_conf and $q_{\det}\geq \tau_{\mathrm{buffer}}$; at most 80 candidates are retained in descending quality order. The same pair-quality matrix is then used in all stages, with stage-specific acceptance thresholds. Thus, OATrack gates detection–track pairs rather than assigning a detection permanently to a high- or low-score pool.

**Figure 3:**
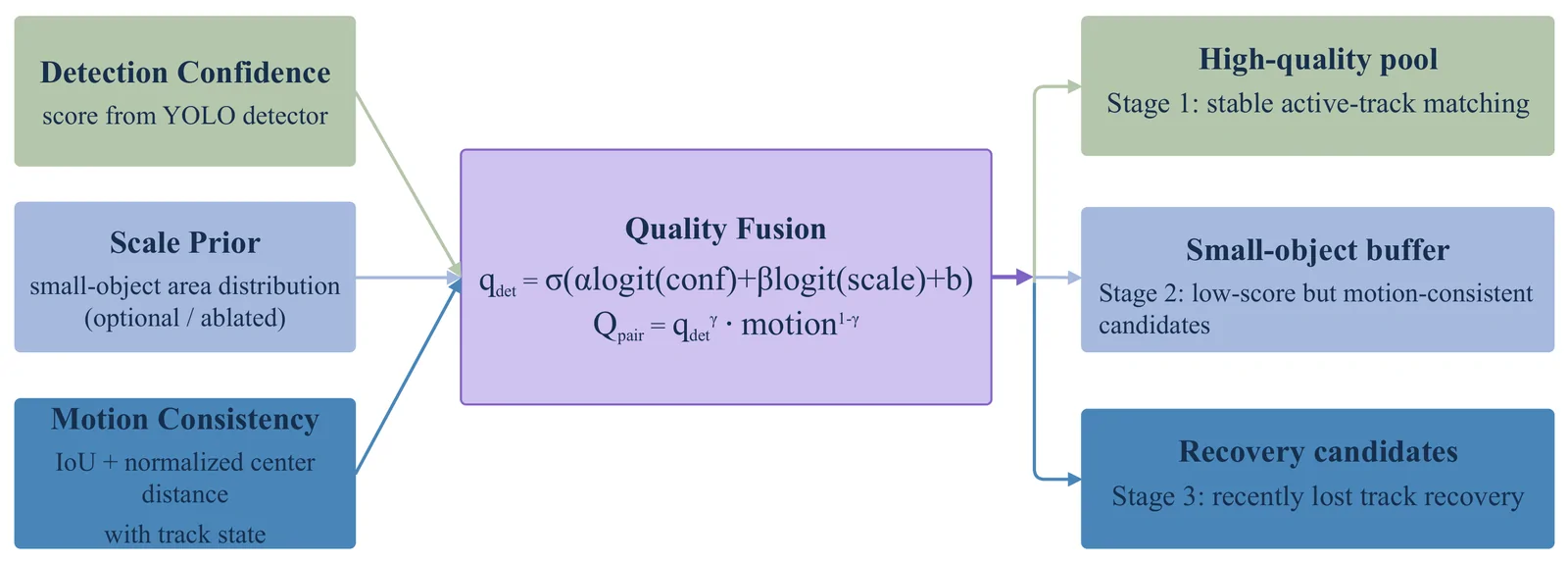
Quality-scoring and stage-routing process. Detection confidence and current-state geometric consistency form the pair quality, with an optional global scale-prior branch for component analysis. Eligible detections enter a shared pair-quality matrix, and the three pool labels summarize their roles as the participating track states and acceptance thresholds in Stages 1–3.

Figure 3 summarizes the quality-scoring and stage-routing process. OATrack first constructs one eligible detection set and a shared pair-quality matrix. The labels “high-quality pool”, “small-object buffer”, and “recovery candidates” indicate the role of candidates as the participating track states and acceptance thresholds change across the three stages. The optional global scale-prior branch is evaluated in the fixed-reference component analysis; the main configuration uses β=0 because confidence–motion quality provides the strongest result among the tested formulations.

### 3.4. Quality-Gated Track Memory

OATrack maintains lightweight state for each trajectory. After a match, the current box, score, class, quality value, and bounded state history are refreshed. A per-track exponentially moving scale prototype is updated only when the detection quality is at least $\max(0.2,0.5\tau_{\mathrm{update}})$. With $\tau_{\mathrm{update}}=0.6$, this update threshold is 0.3. This prevents very low-quality boxes from immediately shifting the auxiliary scale reference.

**Figure 4:**
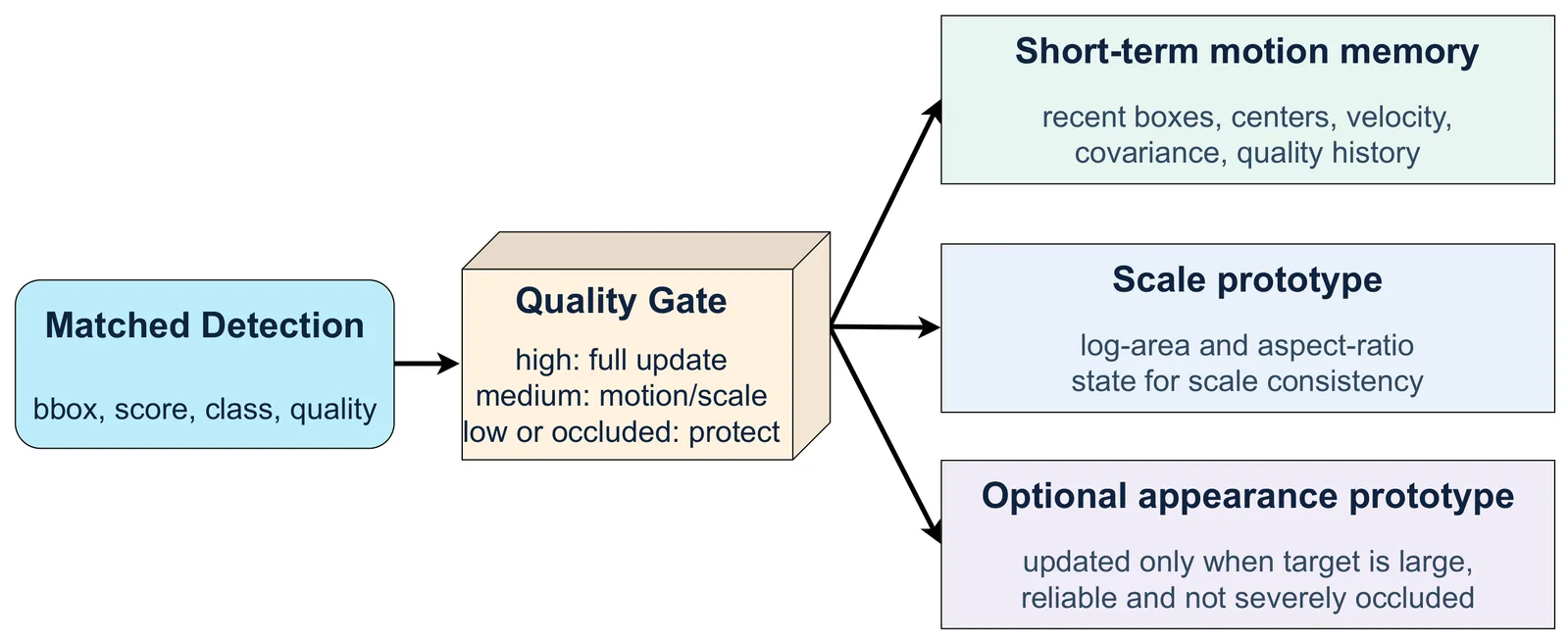
Quality-gated track-state structure. Matching uses the latest observed box and a quality-gated exponentially moving scale prototype. The bounded history stores recent motion and quality statistics, and the appearance prototype provides an interface for future feature-based extensions.

Figure 4 illustrates the quality-gated track-state structure and the protected state used during association.

The implementation contains three state groups. First, the current observed box provides the reference for center-distance and IoU consistency, while a bounded deque stores recent boxes, centers, finite-difference velocities, scales, quality values, and optional covariance fields. Second, an exponentially moving scale prototype maintains log area and aspect ratio and directly modulates pair quality. Third, an appearance prototype provides an optional interface for external embeddings. The main configuration uses the current observed state and protected scale prototype for matching.

The state update follows these rules:

1. Every successful match refreshes the current observed box and appends one state-history record.
2. If quality reaches 0.3, update log area and aspect ratio with momentum 0.9.
3. For a candidate box *d*, compute the track-specific scale consistency
  (7) $$r_{\mathrm{scale}}\left(d,t\right)=0.5+0.5\exp(-|\log A_{d}-m_{A}\left|\right)\exp\left(-\left|\log\frac{r_{d}}{m_{r}}\right|\right),$$
  where $m_{A}$ and $m_{r}$ are the track prototype’s log area and aspect ratio. The assignment score is $\tilde{Q}\left(d,t\right)=Q\left(d,t\right)r_{\mathrm{scale}}\left(d,t\right)$.
4. Do not update or use appearance state when the appearance interface is disabled.

For tiny UAV targets, OATrack adopts the latest observation and protected scale state as compact, reliable association cues. The bounded deque records recent state history, whereas the current pair-quality cost reads the latest box and scale prototype. Consequently, changing the retained history from $L_{s}=5$ to $L_{s}=20$ leaves the discrete assignment metrics unchanged and affects only storage and runtime.

### 3.5. Three-Stage Progressive Assignment

**Figure 5:**
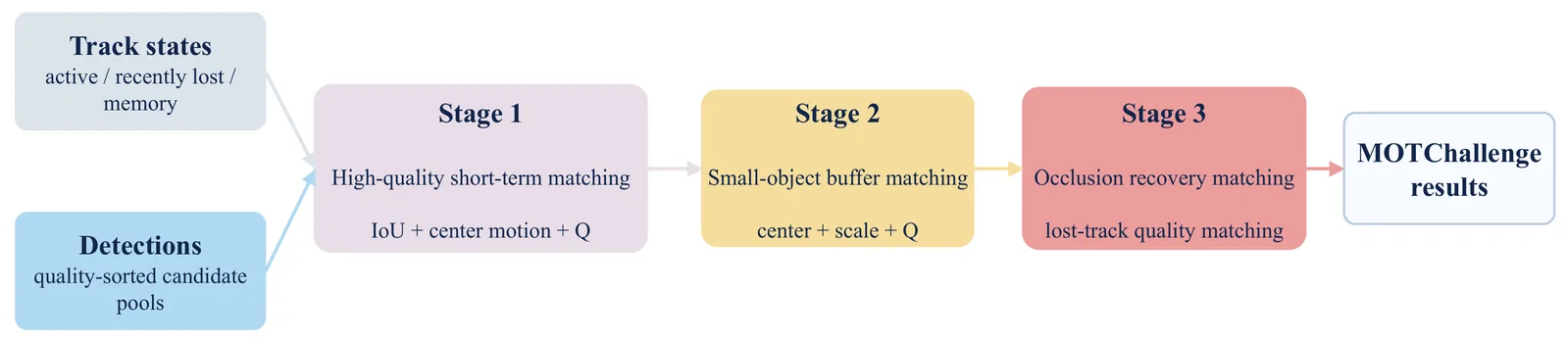
Conceptual roles of the three progressive assignment stages. The labels inside the diagram summarize strict active matching, small-object/unmatched-active retry, and recently lost-track recovery. In the reported implementation, all stages use the same adjusted pair-quality matrix $\tilde{Q}$ and deterministic Hungarian solver; Stage 1 applies threshold 0.70, Stage 2 applies 0.35, and Stage 3 applies 0.25 to their corresponding track sets.

OATrack solves three Hungarian assignments in sequence. It first fixes strict active-track matches, then retries unmatched active tracks and detections at a relaxed threshold, and finally reconnects the remaining detections with recently lost tracks. The term “progressive” refers to these successively relaxed acceptance conditions and the transition from active- to lost-track sets. Figure 5 summarizes the role of each stage; all three stages evaluate the adjusted pair-quality matrix with their corresponding track sets and acceptance thresholds.

As shown in Figure 5, eligible detections are sorted and capped before OATrack constructs $\tilde{Q}$ for active and lost tracks. Each stage minimizes the negative pair-quality matrix with the Hungarian algorithm and rejects assigned pairs below that stage’s threshold.

Stage 1 matches active tracks to all eligible detections using threshold 0.70. Stage 2 uses only unmatched active tracks and detections and lowers the threshold to 0.35; this is the mechanism that retains plausible low-confidence small objects without accepting every low-score box. Stage 3 uses remaining detections and recently lost tracks with threshold 0.25. A successful Stage 3 match restores the original track ID. Otherwise, the track remains lost for at most 30 frames.

The algorithm can be summarized as follows:

1. Input the current detections $D_{t}$, active tracks $T_{a}$, and recently lost tracks $T_{l}$; output updated tracks $T_{t}$.
2. Filter detections using min_conf=0.40, $\tau_{\mathrm{buffer}}=0.20$, and the top-80 cap.
3. Build the adjusted pair-quality matrix $\tilde{Q}$ for active and recently lost tracks.
4. Stage 1: match $T_{a}$ and eligible detections at threshold 0.70.
5. Stage 2: match unmatched active tracks and detections at threshold 0.35.
6. Stage 3: match $T_{l}$ and remaining detections at threshold 0.25, preserving the original ID when successful.
7. Apply the state and prototype updates described in Section 3.4.
8. Initialize an unmatched detection only if $q_{\det}\geq 0.65$; mark unmatched active tracks as lost and remove tracks after 30 lost frames.
9. Output current active tracks and write MOTChallenge-format results.

Compared with a single threshold, this ordering prevents relaxed assignments from displacing strict matches and reserves the final stage for lost-track recovery. Section 4.4 isolates the contribution of the quality formulation, protected state update, and assignment stages at a fixed reference operating point.

## 4. Experiments

### 4.1. Datasets, Conversion, and Evaluation Protocol

Experiments are conducted on VisDrone-MOT and UAVDT. VisDrone-MOT contains multiple object categories, dense occlusion, and large scale variation from urban aerial viewpoints. UAVDT focuses on vehicles captured by UAVs. Table 2 reports the exact splits used after conversion. An object is counted as small when its bounding-box area is below $32^{2}$ pixels, following the commonly used COCO size boundary.

All sequences are converted to MOTChallenge format [27]. The YOLO11m-smallobj detector is trained on the converted VisDrone training split and obtains mAP50 = 0.31576 and mAP50–95 = 0.15793 on VisDrone val. Tracker configurations are developed and reported on VisDrone val. The selected OATrack configuration is then fixed and applied directly to UAVDT without using UAVDT ground truth for detector training or tracker tuning, providing a cross-dataset evaluation on the complete UAVDT test split.

Every method reads the same cached boxes, scores, classes, and frame indices. The YOLO11m-smallobj detector and the unmodified BYTETracker implementation use Ultralytics 8.4.71. In addition to a local score–IoU baseline, we evaluate BYTETracker with its published software defaults (high threshold 0.25, low threshold 0.10, new-track threshold 0.25, buffer 30, match threshold 0.80, and score fusion enabled). The cache adapter feeds standardized detections directly to the official association implementation. This setup reproduces the standard YOLO + ByteTrack tracking route while holding detector output constant.

Evaluation uses TrackEval (commit 12c8791) and reports HOTA, MOTA, IDF1, IDSW, Frag, FP, and FN. The environment uses Python 3.10.19, PyTorch 2.1.2, NumPy 1.26.4, an NVIDIA A40 GPU (NVIDIA Corporation, Santa Clara, CA, USA), and an Intel Xeon Gold 5320 CPU (Intel Corporation, Santa Clara, CA, USA). Cached detections, fixed parameters, Hungarian assignment, and deterministic state updates make the tracking stage deterministic; no random sampling or learned online update is used. The reported accuracy values are therefore deterministic point values rather than averages over stochastic seeds.

Table 3 consolidates the exact OATrack configuration used for the main results. It uses confidence–motion pair quality, the latest observed state, the protected scale prototype, and three-stage assignment; the optional global scale-quality prior, appearance matching, and camera-motion compensation are disabled.

### 4.2. Main Results

Table 4 compares OATrack with the local score–IoU baseline and official Ultralytics ByteTrack under identical cached detections and the same TrackEval protocol. All UAVDT results are computed over the complete 20-sequence test split.

**Figure 6:**
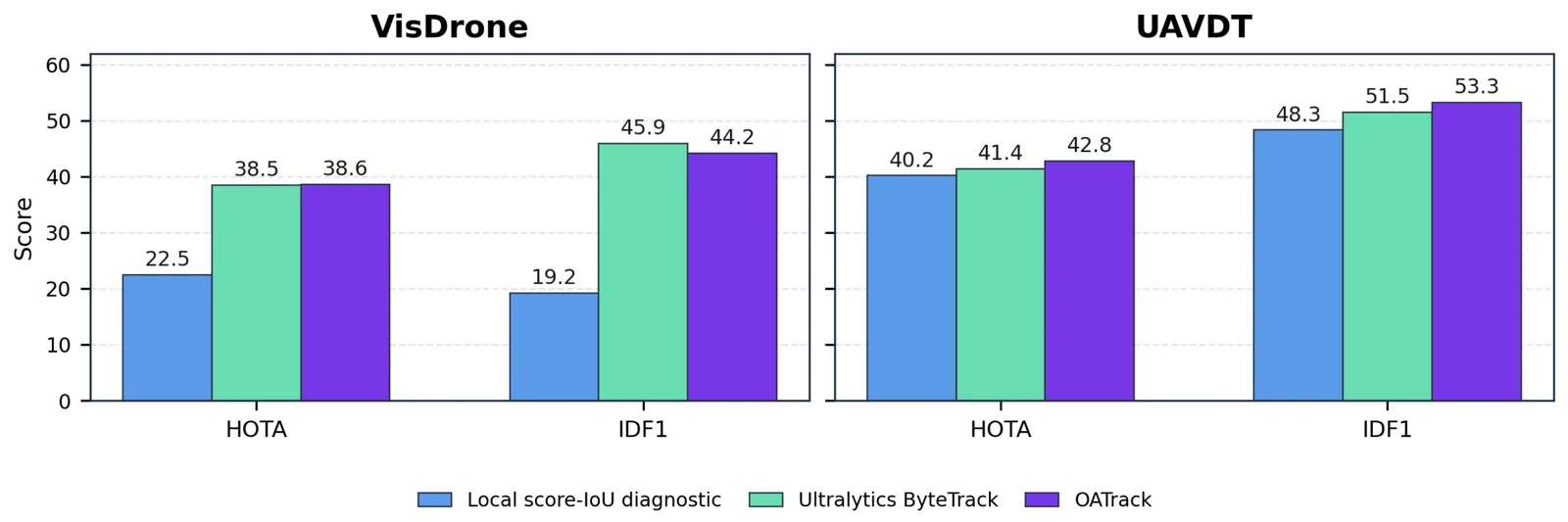
HOTA and IDF1 of the local score–IoU baseline, official Ultralytics ByteTrack, and OATrack under identical cached detections. UAVDT values are computed over the complete 20-sequence test split.

**Figure 7:**
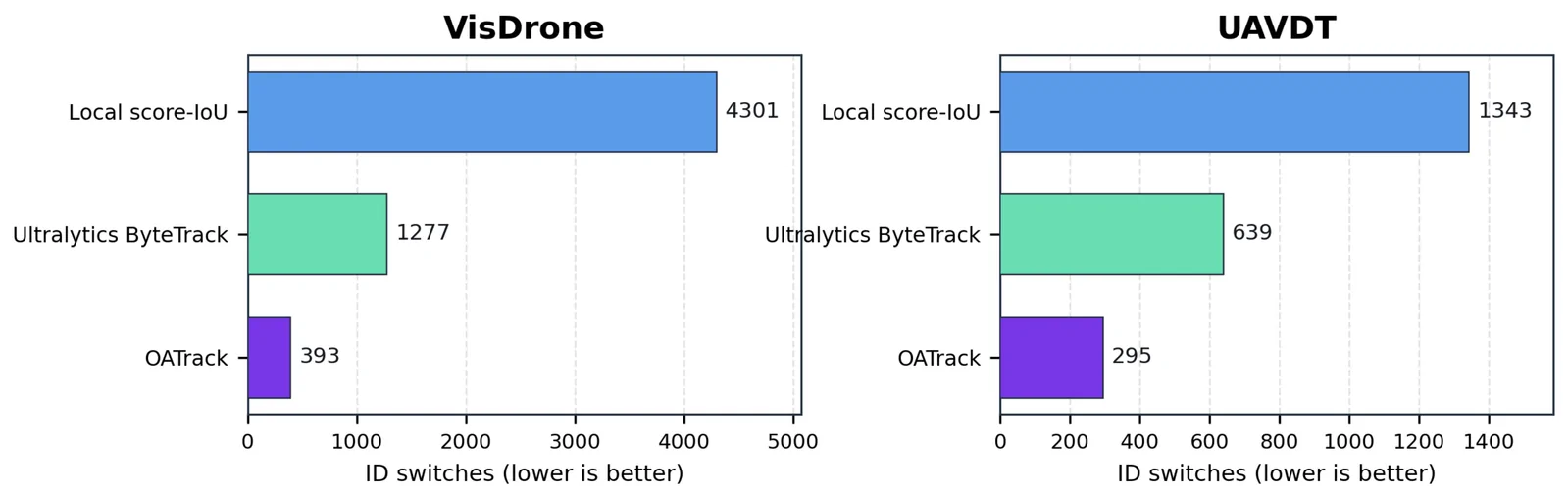
Identity-switch comparison for the same configurations as Figure 6. Lower IDSW indicates stronger identity continuity; UAVDT includes all 20 test sequences.

Table 4 provides the primary fixed-input comparison, including the local score–IoU baseline, official Ultralytics ByteTrack, and OATrack. Figure 6 and Figure 7 visualize the HOTA, IDF1, and identity-switch results from the same table.

On VisDrone val, OATrack reaches HOTA 38.5847 versus 38.4797 for official ByteTrack and substantially strengthens trajectory stability: IDSW decreases by 69.2%, Frag by 29.7%, and FP by 68.8%. Official ByteTrack obtains higher MOTA (31.1017 versus 30.7287) and IDF1 (45.9232 versus 44.2197), while OATrack’s stricter quality gates produce fewer false tracks and identity changes with a higher FN count (73,023 versus 60,787).

On the complete UAVDT test split, OATrack improves HOTA from 41.4345 to 42.7743, MOTA from 7.5162 to 24.8760, and IDF1 from 51.4916 to 53.2829 relative to official ByteTrack. It also reduces IDSW from 639 to 295, Frag from 5301 to 3988, and FP from 183,198 to 89,733. These results demonstrate that the proposed association design generalizes effectively to a vehicle-focused UAV dataset and delivers stronger identity continuity and false-positive suppression under domain shift.

Figure 6 shows that OATrack’s VisDrone HOTA differs from official ByteTrack by 0.105 points and that OATrack achieves higher HOTA and IDF1 on the complete UAVDT split. Figure 7 further shows that OATrack yields the lowest IDSW on both datasets. Together, the figures demonstrate that pair-quality gating and progressive recovery improve identity continuity, with VisDrone HOTA essentially unchanged and UAVDT HOTA increased relative to official ByteTrack.

### 4.3. HOTA Components and Stratified Analysis

Table 5 decomposes HOTA into detection, association, and localization components. OATrack achieves higher association accuracy (AssA) and association recall (AssRe) on both datasets, confirming the effectiveness of pair-quality gating and progressive recovery. It also improves detection precision (DetPr) and localization accuracy (LocA), while official ByteTrack retains higher detection recall (DetRe).

Table 6 further analyzes performance by object size and annotation occlusion. Within each stratum, frame-level Hungarian matching at IoU 0.5 yields match recall, and identity consistency is the fraction of matched observations retaining the modal predicted identity for each ground-truth trajectory. OATrack achieves higher identity consistency in 11 of the 12 dataset–stratum combinations, including all six VisDrone strata, demonstrating robust identity preservation for small and occluded targets. Official ByteTrack obtains higher match recall, indicating that detection recovery remains the principal opportunity for further improvement.

### 4.4. Controlled Component Analysis

To isolate mechanism-level effects independently of operating-point tuning, Table 7 fixes min_conf = 0.60 and varies the quality formulation, protected state update, history retention, and assignment stages. Section 4.5 then evaluates operating-point sensitivity and selects the main q_motion, min_conf = 0.40 configuration.

**Figure 8:**
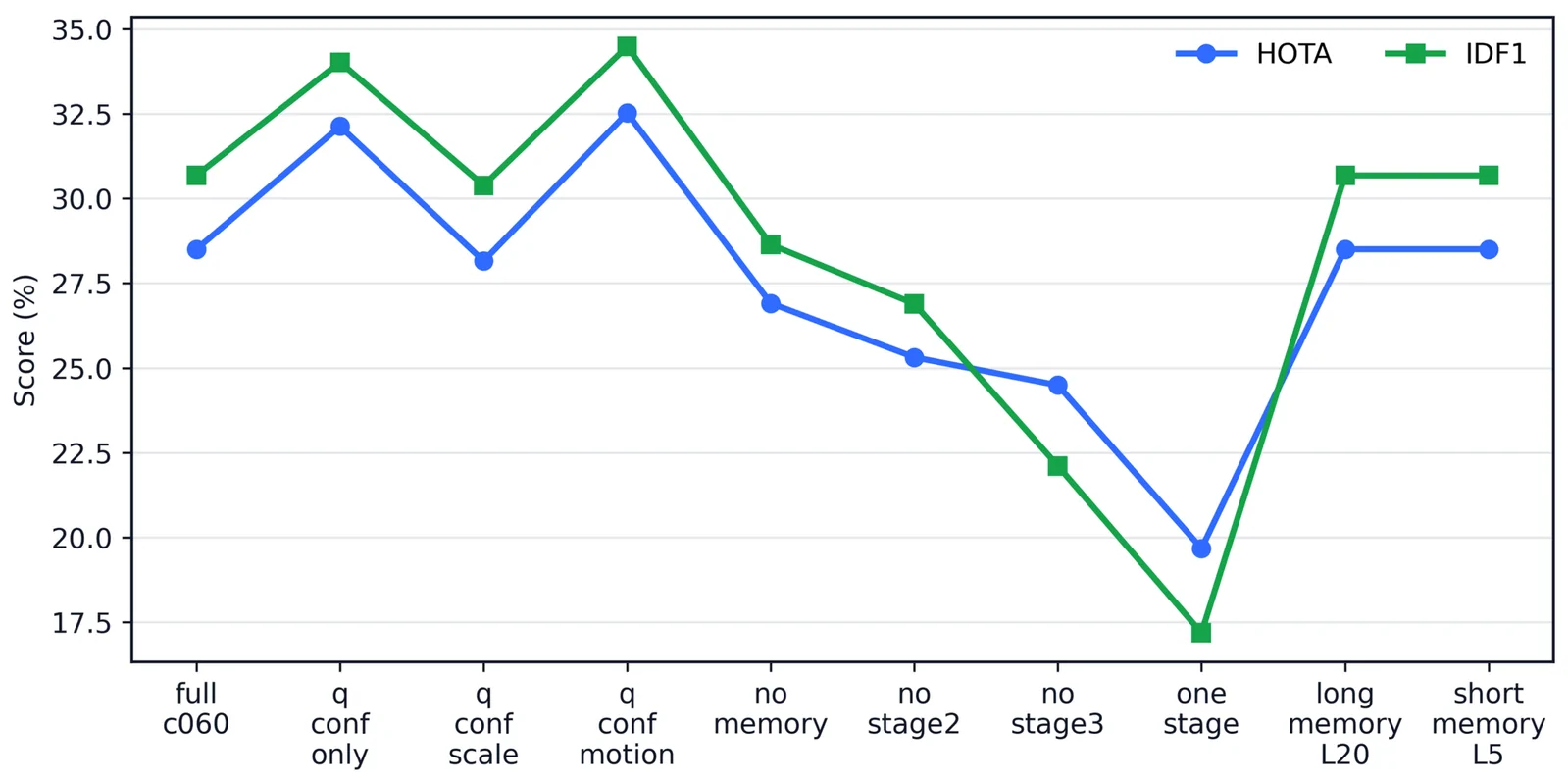
HOTA and IDF1 for the controlled component configurations at the fixed reference operating point min_conf = 0.60. “Long memory L20” and “short memory L5” denote the two retained-history lengths.

Figure 8 visualizes the same fixed-reference configurations. “Full c060” denotes the reference at min_conf = 0.60; “q conf only/scale/motion” denotes the three quality formulations; and “long/short memory” denotes the L=20/5 retained-history settings. Confidence + motion achieves the strongest HOTA and IDF1 among the tested quality formulations. Disabling the protected state update raises IDSW from 584 to 800, while removing Stage 2, removing Stage 3, or using one-stage assignment substantially degrades identity metrics. The L=5 and L=20 settings produce identical accuracy because the current assignment cost uses the latest box and protected scale prototype; the history deque affects retained state records and runtime only.

### 4.5. Parameter Sensitivity and Operating-Point Selection

Table 8 summarizes parameter sensitivity experiments for min_conf, tau_buffer, γ, and association configurations. These experiments analyze how detection threshold, buffer-matching range, quality-fusion weight, and association strategy influence accuracy, identity preservation, FP/FN, and tracker-only FPS.

**Figure 9:**
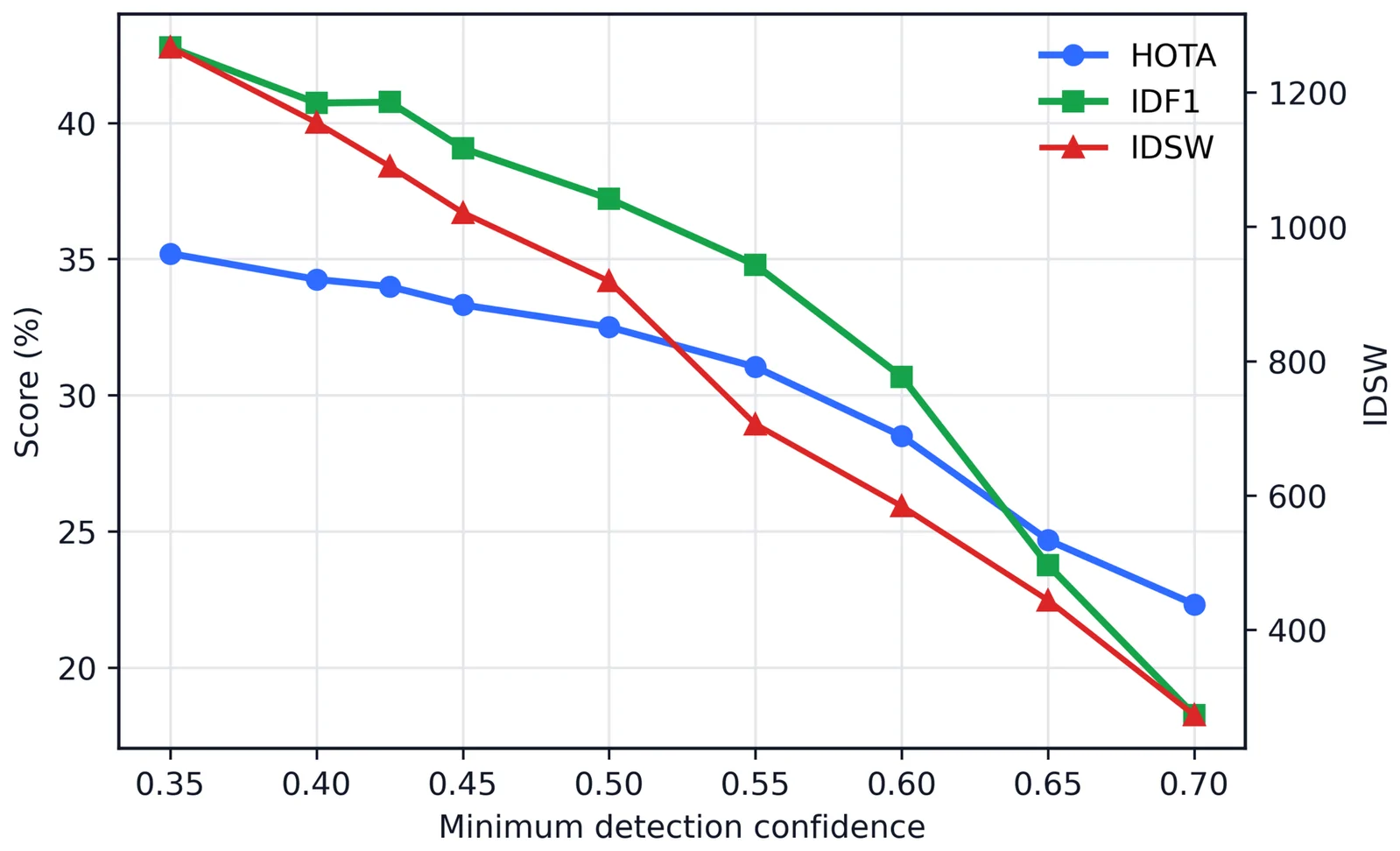
Parameter sensitivity analysis for the detection confidence threshold min_conf. The plot shows how HOTA, IDF1, and IDSW change with the threshold.

**Figure 10:**
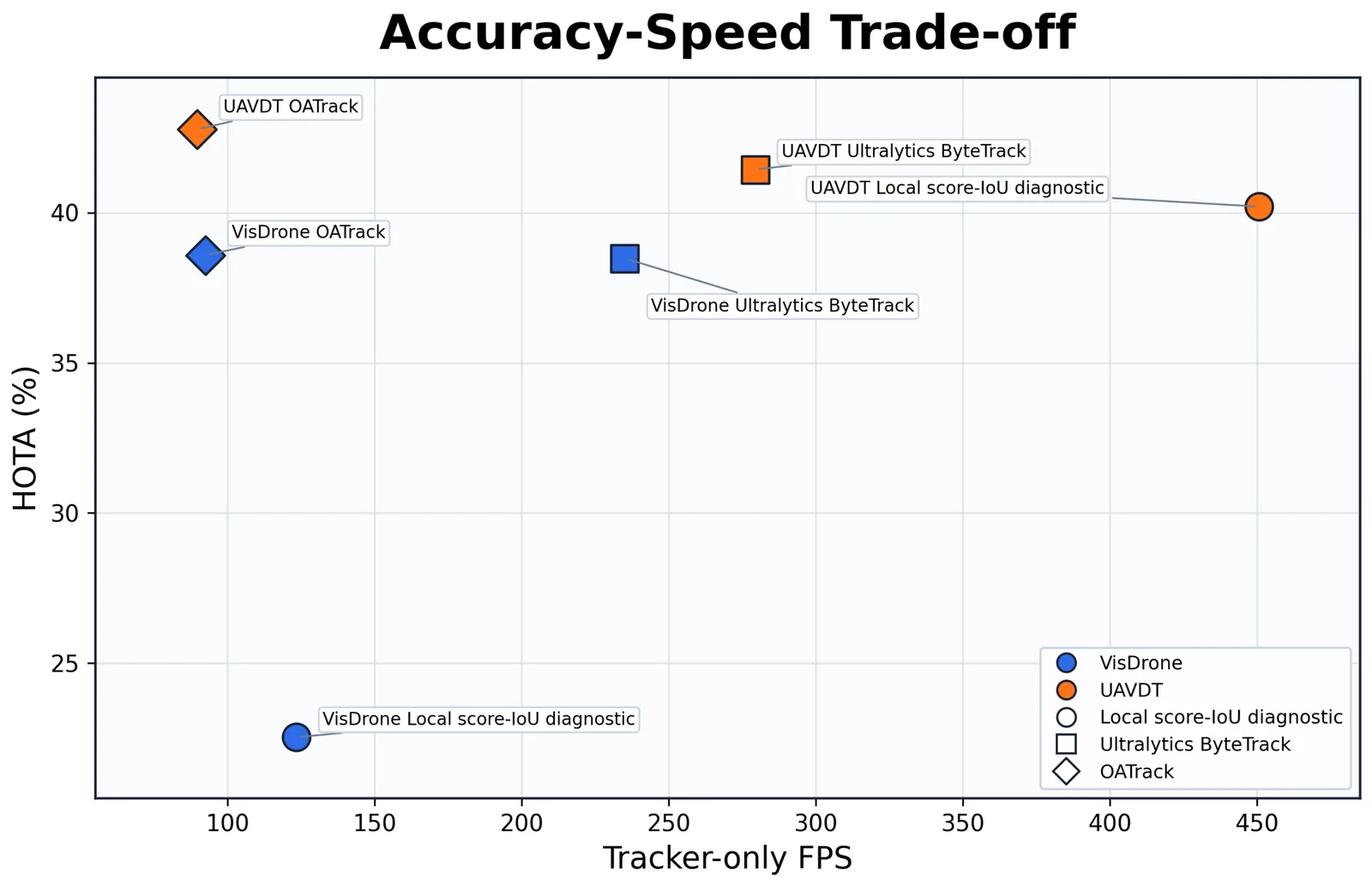
HOTA versus tracker-only FPS for the configurations in Table 4. Colors denote datasets and marker shapes denote methods; FPS measures association-stage throughput.

Figure 9 shows the trends of HOTA, IDF1, and IDSW as min_conf changes, while Table 8 reports the corresponding tracker-only speed. Figure 10 later summarizes the HOTA–speed relationship for the main comparison methods.

The min_conf sweep exposes a recall–precision–speed relationship. Lower thresholds introduce more candidates and improve HOTA/IDF1, while increasing FP and association cost. The limited change over $\tau_{\mathrm{buffer}}=0.10$–0.30 indicates stable performance within the tested range. At γ=0.90, IDSW rises sharply, showing that over-weighting detection confidence weakens the motion constraint. The q_motion configurations also run faster than the loose configurations in Table 8 (92.50–105.03 versus 57.52–69.26 FPS). The q_motion, mc=0.40, setting provides the strongest HOTA and IDF1 and is applied unchanged to UAVDT.

### 4.6. Per-Sequence Results and Runtime

Table 9 gives the complete seven-sequence VisDrone comparison. OATrack lowers IDSW on six of seven sequences and achieves higher HOTA on three sequences, including gains of 2.08, 4.63, and 3.60 points on uav0000117, uav0000305, and uav0000339, respectively. These results show that OATrack’s identity-preserving association is particularly effective in several crowded and small-object sequences.

Table 10 reports all 20 UAVDT test sequences. OATrack achieves higher HOTA on 12 sequences and lower IDSW on 17, with one tie. Particularly large HOTA gains occur on M0801, M0802, M1303, and M1101. M1009 remains the most challenging sequence because the VisDrone-trained detector provides very few reliable candidates under the UAVDT domain shift.

Runtime is separated into detector-only, tracker-only, and serial pipeline estimates in Table 11. Detector timing covers all 2846 VisDrone val frames at 1536 pixels and batch size 4 and includes data loading, preprocessing, inference, and postprocessing wall time. The serial estimate $F_{e2e}=1/\left(1/F_{det}+1/F_{trk}\right)$ represents detector and tracker execution in sequence; video decoding, visualization, telemetry, and platform scheduling are outside this measurement.

Figure 10 visualizes HOTA against tracker-only FPS for the configurations in Table 4. Marker shapes distinguish the local score–IoU baseline, official Ultralytics ByteTrack, and OATrack, while colors distinguish the two datasets. The scatter directly compares association accuracy and association-stage throughput under identical detector inputs.

OATrack processes the association stage at 92.50 FPS on VisDrone and 89.64 FPS on UAVDT, supporting real-time tracking association. Official ByteTrack is faster at 235.04 and 279.33 FPS, respectively, whereas OATrack keeps VisDrone HOTA within 0.11 points of ByteTrack, raises UAVDT HOTA by 1.34 points, and substantially reduces identity switches. In the measured high-resolution VisDrone pipeline, detector inference at 6.1597 FPS dominates total runtime; the serial estimates are 6.0024 FPS for ByteTrack and 5.7751 FPS for OATrack, a difference of approximately 3.8%. Faster detection, pipeline overlap, and platform-specific optimization can therefore improve end-to-end throughput for future onboard deployment.

### 4.7. Comparison with Published Methods

Table 12 positions OATrack within recent UAV and traffic-scene MOT research using the MOTA, IDF1, IDSW, FP, and FN values reported by the corresponding papers. Because these methods use different detectors, training splits, annotation versions, confidence thresholds, and evaluation implementations, the table provides the broad literature context, while Table 4, Table 5 and Table 6 provide the controlled fixed-input evidence.

Methods with end-to-end Transformer detectors, dedicated detectors, or more complex training strategies report strong MOTA and IDF1 in their respective protocols. With a unified YOLO detection interface, OATrack concentrates computation on pair-quality gating and progressive association, prioritizing identity continuity within a compact association design.

On VisDrone val, OATrack reports IDSW 393, which is below the values reported by GAO-Tracker and ETDMOT in their respective protocols. On UAVDT, its IDSW 295 is comparable to SFTrack (284) and SocialTrack (276). The external comparison highlights OATrack’s identity-preservation capability, while the fixed-input results in Table 4, Table 5 and Table 6 quantify its association behavior under a unified detector and evaluation protocol.

### 4.8. Visualization and Error Analysis

**Figure 11:**
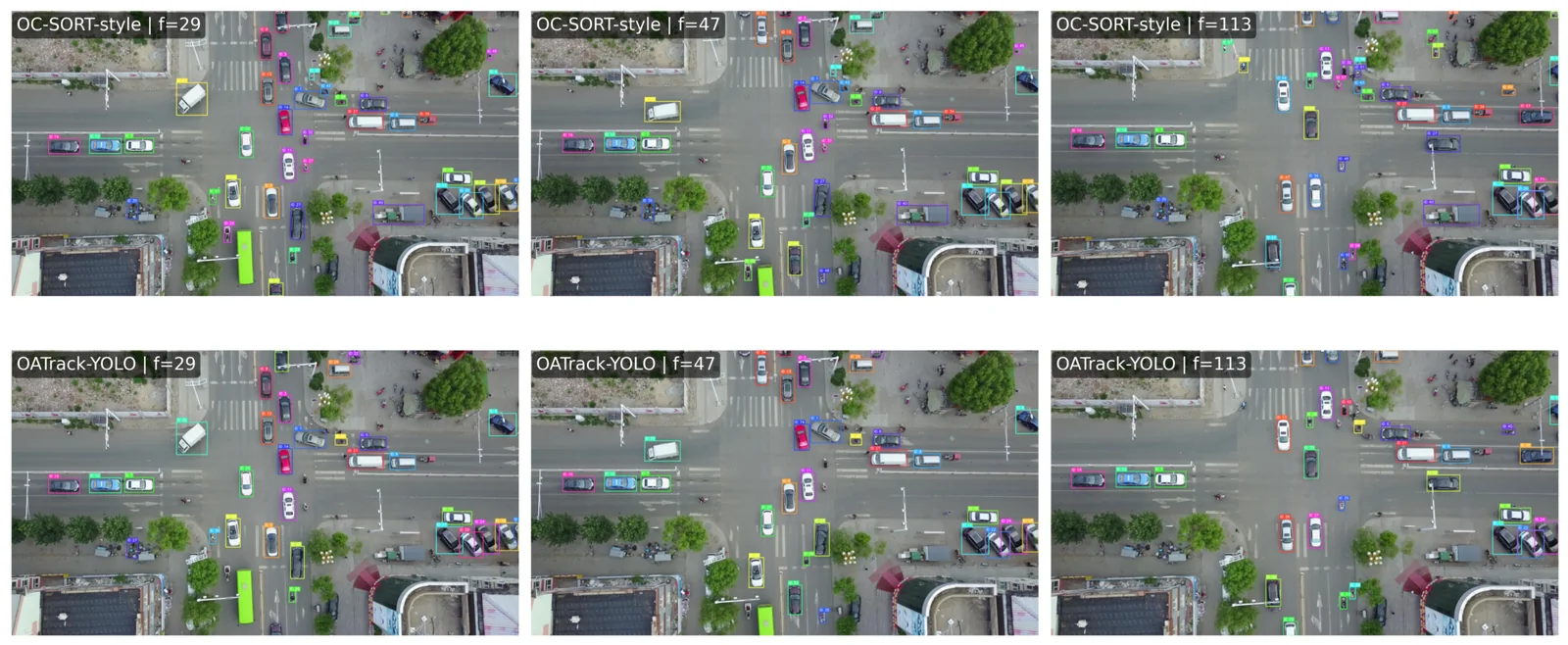
VisDrone qualitative comparison between the local motion-parameter configuration (“OC-SORT-style” in the panel) and OATrack (“OATrack-YOLO” in the panel). Colored bounding boxes and labels distinguish tracker-assigned identities; the colors do not encode object categories.

**Figure 12:**
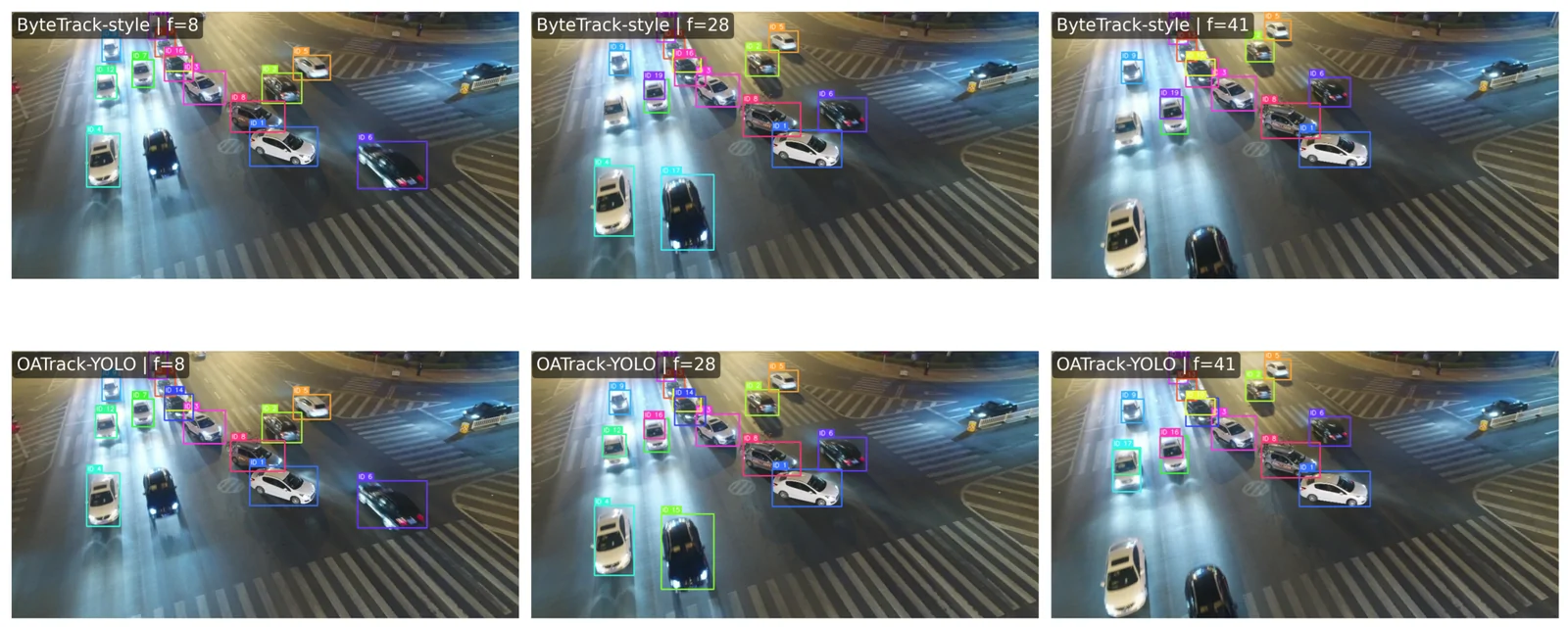
UAVDT qualitative comparison between the local score–IoU configuration (“ByteTrack-style” in the panel) and OATrack (“OATrack-YOLO” in the panel). Colored bounding boxes and labels distinguish tracker-assigned identities; the colors do not encode object categories.

**Figure 13:**
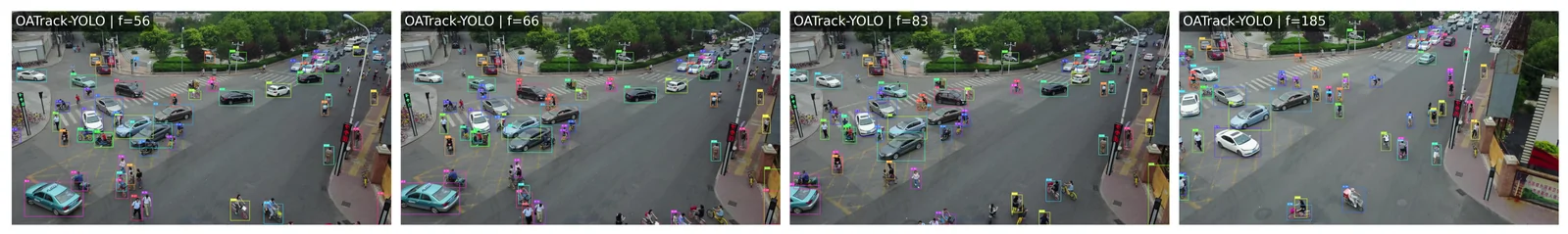
OATrack visualization in a dense VisDrone small-object scene. Colored bounding boxes and labels distinguish tracker-assigned identities; the colors do not encode object categories.

**Figure 14:**
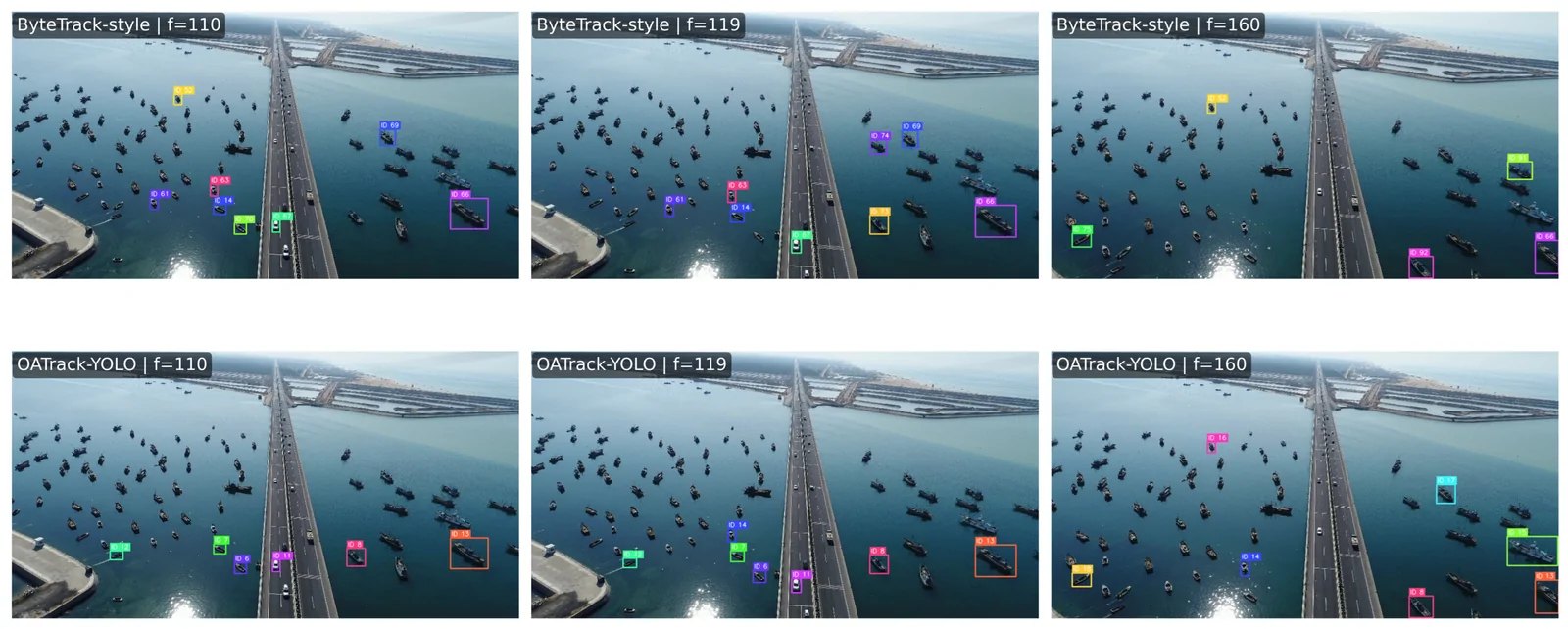
Challenging UAVDT cases involving background noise, tiny targets, and occlusion. Colored bounding boxes and labels distinguish tracker-assigned identities; the colors do not encode object categories.

Representative sequences from VisDrone-MOT and UAVDT are visualized to examine OATrack’s association behavior. The compact labels embedded in the panels identify the evaluated configurations: “OC-SORT-style” denotes the local motion-parameter configuration, “ByteTrack-style” denotes the local score–IoU configuration, and “OATrack-YOLO” denotes OATrack with the shared YOLO detections. Figure 11 and Figure 12 show successful comparisons on VisDrone-MOT and UAVDT, respectively; Figure 13 presents OATrack outputs in a dense small-object scene; and Figure 14 illustrates challenging UAVDT cases. The visualizations show stable identities through crowding, viewpoint changes, and short occlusions.

Figure 12 shows a successful qualitative comparison on UAVDT. Under viewpoint changes, short vehicle spacing, and partial occlusion, OATrack maintains continuous vehicle identities after being configured on VisDrone-MOT, demonstrating cross-dataset transfer without UAVDT-specific tracker tuning.

Figure 14 shows challenging cases. When targets are extremely small, detections are continuously missing, or persistent low-score distractors appear in complex backgrounds, association remains difficult. Improving small-object detector recall and reliability is therefore the direct route to supplying stronger candidates for the subsequent association stage.

Overall, the qualitative results support the quantitative evaluation: OATrack preserves identities effectively across crowded and occluded scenes, while extremely small targets and prolonged detection gaps remain the principal failure cases.

## 5. Summary and Future Work

### 5.1. Summary

This paper presented OATrack, an integrated association framework for UAV small-object MOT. Detection–track pair quality combines detection reliability with geometric consistency, quality-gated updates protect the track-specific scale prototype from unreliable observations, and three progressively relaxed assignments handle strict active matching, unmatched-active retry, and recently lost-track recovery. The standardized YOLO detection interface further enables deterministic replay and reproducible fixed-input evaluation.

Under identical detector outputs, OATrack reaches HOTA 38.5847 versus 38.4797 for official Ultralytics ByteTrack on VisDrone val and reduces IDSW from 1277 to 393. On the complete 20-sequence UAVDT test split, it improves HOTA from 41.4345 to 42.7743 and IDF1 from 51.4916 to 53.2829 while reducing IDSW from 639 to 295. HOTA decomposition and stratified analysis further confirm stronger association consistency and identity preservation across object sizes and occlusion levels.

### 5.2. Future Work

The results identify several promising directions for further development. Independent multi-dataset testing can extend the current VisDrone-to-UAVDT evaluation, while recall-aware adaptive gates can improve the recovery of very small and domain-shifted targets. UAV-specific appearance features and camera-motion compensation can provide additional cues during prolonged occlusion and rapid viewpoint change. Future work will also combine accelerated detection with pipeline-level optimization and platform-specific latency and power measurements for onboard deployment.

## Figures and Tables

**Table 1:** Standardized fields stored in each detection-cache record.

Field	Stored Information
frame_id	Frame index of the detection.
bbox_xyxy	Upper-left and lower-right bounding-box coordinates.
bbox_xywh	Upper-left coordinate, width, and height of the bounding box.
score	Detection confidence.
class_id	Object category index.
image_size	Image width and height.
optional_embedding	Optional appearance feature for association strategies that use appearance information.

**Table 2:** Dataset composition used in this study. Annotation and track counts were computed from the converted ground-truth files; the small-object ratio uses the $32^{2}$-pixel area boundary.

Dataset	Split	Sequences	Frames	Annotations	Tracks	Small (%)	Role
VisDrone-MOT	train	56	24,201	1,098,849	7290	24.97	detector training
VisDrone-MOT	val	7	2846	113,145	751	19.31	tracker development/reporting
UAVDT	test	20	16,592	375,884	1287	74.87	cross-dataset evaluation

**Table 3:** Final OATrack configuration used for the reported main results.

Item	Value	Item	Value
Minimum confidence min_conf	0.40	Detection-quality gate $\tau_{\mathrm{buffer}}$	0.20
New-track threshold $\tau_{\mathrm{high}}$	0.65	Maximum detections/frame	80
Quality weight γ	0.60	Motion decay λ	0.50
Stage-1 threshold	0.70	Stage-2 threshold	0.35
Stage-3 threshold	0.25	Maximum lost duration	30 frames
State-update threshold $\tau_{\mathrm{update}}$	0.60	Scale-update threshold	0.30
Scale EMA momentum	0.90	Retained state history $L_{s}$	10
Global scale prior (α,β,b)	(1,0,0); disabled	Appearance/ReID	disabled
Camera-motion compensation	disabled	Assignment	Hungarian, deterministic

**Table 4:** Main tracking results under identical YOLO11m-smallobj cached detections and the same TrackEval protocol. UAVDT includes all 20 test sequences. FPS is tracker-only.

Dataset/Split	Method	HOTA	MOTA	IDF1	IDSW	Frag	FP	FN	FPS
VisDrone val	Local score–IoU baseline *	22.5311	14.7174	19.1860	4301	792	1699	90,493	123.39
VisDrone val	Ultralytics ByteTrack	38.4797	31.1017	45.9232	1277	2104	15,891	60,787	235.04
VisDrone val	OATrack	38.5847	30.7287	44.2197	393	1479	4961	73,023	92.50
UAVDT test	Local score–IoU baseline *	40.2032	20.9780	48.3239	1343	7146	97,662	198,026	450.60
UAVDT test	Ultralytics ByteTrack	41.4345	7.5162	51.4916	639	5301	183,198	163,795	279.33
UAVDT test	OATrack	42.7743	24.8760	53.2829	295	3988	89,733	192,351	89.64

* The local score–IoU baseline provides a simple fixed-input association reference. Larger HOTA, MOTA, IDF1, and FPS are better; smaller IDSW, Frag, FP, and FN are better.

**Table 5:** HOTA decomposition under identical cached detections. All entries are percentages.

Dataset	Method	HOTA	DetA	AssA	DetRe	DetPr	AssRe	AssPr	LocA
VisDrone val	Ultralytics ByteTrack	38.480	32.413	47.176	37.314	61.860	51.003	77.458	79.023
VisDrone val	OATrack	38.585	27.329	55.355	29.021	72.835	61.887	73.034	81.670
UAVDT test	Ultralytics ByteTrack	41.435	29.971	57.581	45.452	43.221	65.303	76.465	81.378
UAVDT test	OATrack	42.774	31.493	58.343	39.828	54.784	65.655	75.799	82.519

**Table 6:** Stratified recall and identity-consistency results (%). “None”, “moderate”, and “severe” follow the converted benchmark occlusion annotations.

		Small		Medium		Large		None		Moderate		Severe	
Dataset	Method	R	IC	R	IC	R	IC	R	IC	R	IC	R	IC
VisDrone	ByteTrack	22.45	84.91	47.99	76.22	76.88	87.76	64.28	81.09	37.00	74.58	10.49	86.79
VisDrone	OATrack	9.77	92.51	36.76	89.76	71.48	95.15	53.66	93.26	25.44	87.43	3.76	98.95
UAVDT	ByteTrack	48.16	92.86	84.82	95.33	40.02	91.46	56.79	93.50	15.42	97.41	59.22	91.15
UAVDT	OATrack	39.50	96.42	80.57	96.25	33.87	94.58	49.11	96.31	10.84	92.07	52.14	92.49

**Table 7:** Controlled component analysis on VisDrone val at the fixed reference operating point min_conf = 0.60.

Setting	HOTA	MOTA	IDF1	IDSW	Frag	FP	FN	FPS
Reference configuration (mc=0.60)	28.5017	20.2121	30.6798	584	2179	2236	87,456	164.03
Confidence only	32.1279	22.6099	34.0200	612	1843	2156	84,795	183.21
Confidence + optional global scale	28.1569	19.7578	30.3872	746	2165	2206	87,838	150.55
Confidence + motion	32.5203	23.1517	34.4817	427	2077	2259	84,264	187.29
Without protected state update	26.9094	20.0327	28.6413	800	2219	2264	87,415	197.86
Retained history L=5	28.5017	20.2121	30.6798	584	2179	2236	87,456	165.42
Retained history L=20	28.5017	20.2121	30.6798	584	2179	2236	87,456	167.27
w/o Stage 2	25.3149	16.9844	26.8905	4106	2696	2211	87,611	135.80
w/o Stage 3	24.4925	18.1369	22.1124	2007	2011	2165	88,452	87.44
one-stage association	19.6795	15.4298	17.1881	2735	2180	2088	90,864	71.64

**Table 8:** Hyperparameter sensitivity results. The table reports the influence of key thresholds and association weights on HOTA, IDF1, IDSW, FP/FN, and tracker-only FPS.

Parameter	Setting	HOTA	MOTA	IDF1	IDSW	Frag	FP	FN	FPS
min_conf	0.35	35.2011	28.7816	42.7704	1267	2889	11,533	67,780	45.25
min_conf	0.40	34.2516	28.5333	40.7288	1155	2844	8984	70,722	54.76
min_conf	0.425	33.9896	28.1515	40.7620	1090	2810	7924	72,279	61.06
min_conf	0.45	33.3158	27.5982	39.0652	1021	2779	6848	74,050	67.93
min_conf	0.50	32.5070	25.8041	37.2139	919	2637	5000	78,030	85.25
min_conf	0.55	31.0368	23.5459	34.7835	706	2376	3493	82,305	116.03
min_conf	0.60	28.5017	20.2121	30.6798	584	2179	2236	87,456	166.58
min_conf	0.65	24.6809	15.5084	23.7617	444	1885	1296	93,858	264.50
min_conf	0.70	22.3231	10.7013	18.2543	273	1171	740	100,024	493.51
tau_buffer	0.10	28.5121	20.2307	30.7060	584	2197	2237	87,434	160.95
tau_buffer	0.20	28.5017	20.2121	30.6798	584	2179	2236	87,456	165.97
tau_buffer	0.30	28.4219	20.1193	30.5925	574	2155	2217	87,590	172.54
γ	0.30	28.8871	20.4746	31.2991	578	2163	2222	87,179	155.01
γ	0.60	28.5017	20.2121	30.6798	584	2179	2236	87,456	166.12
γ	0.90	24.6058	19.2046	24.8397	1145	2258	2249	88,022	186.77
association	loose, mc = 0.45	33.0521	27.8996	38.1793	1160	2815	7068	73,350	69.26
association	loose, mc = 0.40	33.5940	28.9611	39.4990	1324	2908	9338	69,715	57.52
association	medium, mc = 0.45	33.2774	27.8430	38.5852	1133	2811	7008	73,501	69.76
association	q_motion, mc = 0.45	38.0780	29.5435	43.3659	398	1587	4468	74,852	105.03
association	q_motion, mc = 0.40	38.5847	30.7287	44.2197	393	1479	4961	73,023	92.50
association	q_conf, mc = 0.45	37.9398	28.5430	43.1033	568	1313	3707	76,575	108.91

**Table 9:** Per-sequence VisDrone val results under identical cached detections. UBT denotes official Ultralytics ByteTrack; OA denotes OATrack.

Sequence	HOTA UBT	HOTA OA	IDF1 UBT	IDF1 OA	IDSW UBT	IDSW OA
uav0000086_00000_v	42.073	38.874	55.587	50.254	144	26
uav0000117_02622_v	29.111	31.190	32.075	35.228	321	36
uav0000137_00458_v	42.517	43.078	52.656	50.389	521	167
uav0000182_00000_v	32.666	30.147	42.350	36.187	141	59
uav0000268_05773_v	34.045	33.582	32.498	31.223	4	2
uav0000305_00000_v	50.373	55.001	59.370	62.534	39	80
uav0000339_00001_v	36.150	39.750	39.499	43.082	107	23

**Table 10:** Per-sequence UAVDT test results for all 20 sequences. UBT denotes official Ultralytics ByteTrack; OA denotes OATrack.

Seq.	H-U	H-O	I-U	I-O	S-U	S-O	Seq.	H-U	H-O	I-U	I-O	S-U	S-O
M0203	55.41	56.93	66.52	67.09	21	15	M0802	46.52	52.53	55.40	65.77	23	12
M0205	65.59	68.03	77.76	79.76	19	6	M1001	32.23	27.86	41.99	37.74	43	7
M0208	8.48	6.27	6.89	4.36	4	0	M1004	11.67	5.51	16.70	4.52	4	1
M0209	47.31	50.80	59.74	66.71	56	14	M1007	34.07	36.49	42.16	48.02	10	8
M0403	66.97	70.22	84.67	88.85	14	17	M1009	3.30	0.40	4.85	0.31	7	0
M0601	16.52	16.07	20.52	21.07	112	13	M1101	31.55	35.17	40.35	47.58	22	7
M0602	24.61	17.59	36.25	23.55	60	20	M1301	46.64	48.90	65.58	69.65	35	11
M0606	32.14	36.61	36.52	43.86	39	27	M1302	40.88	39.99	56.30	54.42	55	41
M0701	23.54	19.87	32.48	25.04	67	54	M1303	57.16	63.53	71.11	79.28	9	12
M0801	64.55	75.08	73.59	86.73	3	3	M1401	50.15	43.47	67.75	58.31	36	27

H: HOTA; I: IDF1; S: IDSW; U: UBT; O: OATrack.

**Table 11:** Runtime decomposition on the NVIDIA A40. Detector-only timing is common because cached detections are identical.

Association Method	Detector-Only FPS	Tracker-Only FPS	Serial Estimate FPS
Ultralytics ByteTrack	6.1597	235.04	6.0024
OATrack	6.1597	92.50	5.7751

**Table 12:** Reported metrics of recent UAV/traffic-scene MOT methods. Detector and evaluation protocols follow the corresponding publications.

Dataset/Split	Method	Detector/Protocol	MOTA	IDF1	IDSW	FP	FN
VisDrone val	OATrack (ours)	YOLO11m-smallobj cache	30.73	44.22	393	4961	73,023
VisDrone-MOT	GAO-Tracker [28]	Holistic Trans-Det	38.8	54.3	972	6883	10,204
VisDrone-MOT	ETDMOT [29]	Transformer detector	38.9	65.7	981	6793	10,093
UAVDT test (20 seq.)	OATrack (ours)	YOLO11m-smallobj cache	24.88	53.28	295	89,733	192,351
UAVDT (reported protocol)	SFTrack [30]	original-paper protocol	55.3	71.3	284	49,946	117,224
UAVDT	SocialTrack [21]	SOFEPNet	64.6	76.1	276	31,133	89,146
UAVDT	GAO-Tracker [28]	Holistic Trans-Det	61.7	67.9	1216	24,915	59,640
UAVDT	ETDMOT [29]	Transformer detector	61.9	68.1	1278	21,478	51,238

## Data Availability

The VisDrone-MOT and UAVDT datasets analyzed in this study are publicly available from their respective benchmark websites. The generated detection cache, converted annotations, and tracking results used in this study can be made available from the corresponding author upon reasonable request.
